# Clinical characteristics and risk factors of acute lymphoblastic leukemia in children with severe infection during maintenance treatment

**DOI:** 10.1002/cam4.6495

**Published:** 2023-09-28

**Authors:** Tiantian Yin, Juan Han, Jinjin Hao, Hui Yu, Yining Qiu, Jiawei Xu, Yun Peng, Xiaoyan Wu, Runming Jin, Fen Zhou

**Affiliations:** ^1^ Department of Pediatrics, Union Hospital, Tongji Medical College Huazhong University of Science and Technology Wuhan China

**Keywords:** acute lymphoblastic leukemia, children, maintenance phase, risk factors, severe infection

## Abstract

**Background:**

Infection is the most common adverse event of acute lymphoblastic leukemia (ALL) treatment and is also one of the main causes of death.

**Methods:**

To investigate the clinical characteristics and risk factors of severe infections during the maintenance phase of ALL treatment, we conducted a retrospective study.

**Results:**

A total of 181 children were eligible and 46 patients (25.4%) suffered from 51 events of severe infection, most of which occurred in the first half year of the maintenance phase (52.9%). The most common infection was pulmonary infection (86.3%) followed by bloodstream infection (19.6%). The main symptoms of ALL patients with pulmonary infection were fever, cough, and shortness of breath. The main manifestations of computer tomography (CT) were ground glass shadow (56.8%), consolidation shadow (27.3%), and streak shadow (25%). Multivariate binary logistic regression analysis showed that agranulocytosis, agranulocytosis ≥7 days, anemia, and low globulin level were independent risk factors for severe infection during the maintenance phase (all *p* < 0.05).

**Conclusions:**

Taken together, blood routine examinations and protein levels should be monitored regularly for ALL patients in the maintenance phase, especially in the first 6 months. For ALL patients with risk factors, preventive anti‐infective or supportive therapies can be given as appropriate to reduce the occurrence of severe infections.

## 1 INTRODUCTION

Acute lymphoblastic leukemia (ALL) is the most common malignant tumor in children, with an incidence of 3–5 per 100,000, accounting for 30% of all malignant tumors and 80% of childhood leukemia.^1^, ^2^, ^3^, ^4^ Combined chemotherapy is still the main treatment for ALL. Although the overall survival (OS) of ALL patients has significantly improved, some still succumb to infections or recurrence.^1^, ^5^ Infection mainly occurs due to the effect of tumors and chemotherapy, immunosuppression, and their susceptibility to bacteria, fungi, and viruses.^6^ Severe infection even becomes the main cause of death in children with ALL.^7^ In published studies, infection‐related mortality during childhood treatment ALL ranged from 1.7% to 2.4%.^8^ Previous studies have reported that severe infection occurs mostly during or after intensive chemotherapy phases, induction, and reinduction.^8^, ^9^ However, severe infections during the maintenance phase should also be closely monitored.

Maintenance therapy is one of the main components of childhood ALL treatment.^10^ Mercaptopurine and methotrexate are the core drugs used in the maintenance treatment.^11^ In our study, the maintenance phase of the CCCG‐ALL‐2015 protocol lasted 1.75–2 years.^12^ The purpose is to eliminate leukemia cells gradually and prevent relapse.^13^ Although children have achieved complete remission and gradual immune function recovery during the maintenance phase, many still exhibit immune dysfunction, making them prone to infections.^14^, ^15^ In this study, we conducted a retrospective analysis of the clinical characteristics and risk factors associated with severe infections in children with ALL during the maintenance treatment phase. We also assessed the impact of severe infections on patient outcomes.

## 2 METHODS

### 2.1 Study design and participants

**FIGURE 1:**
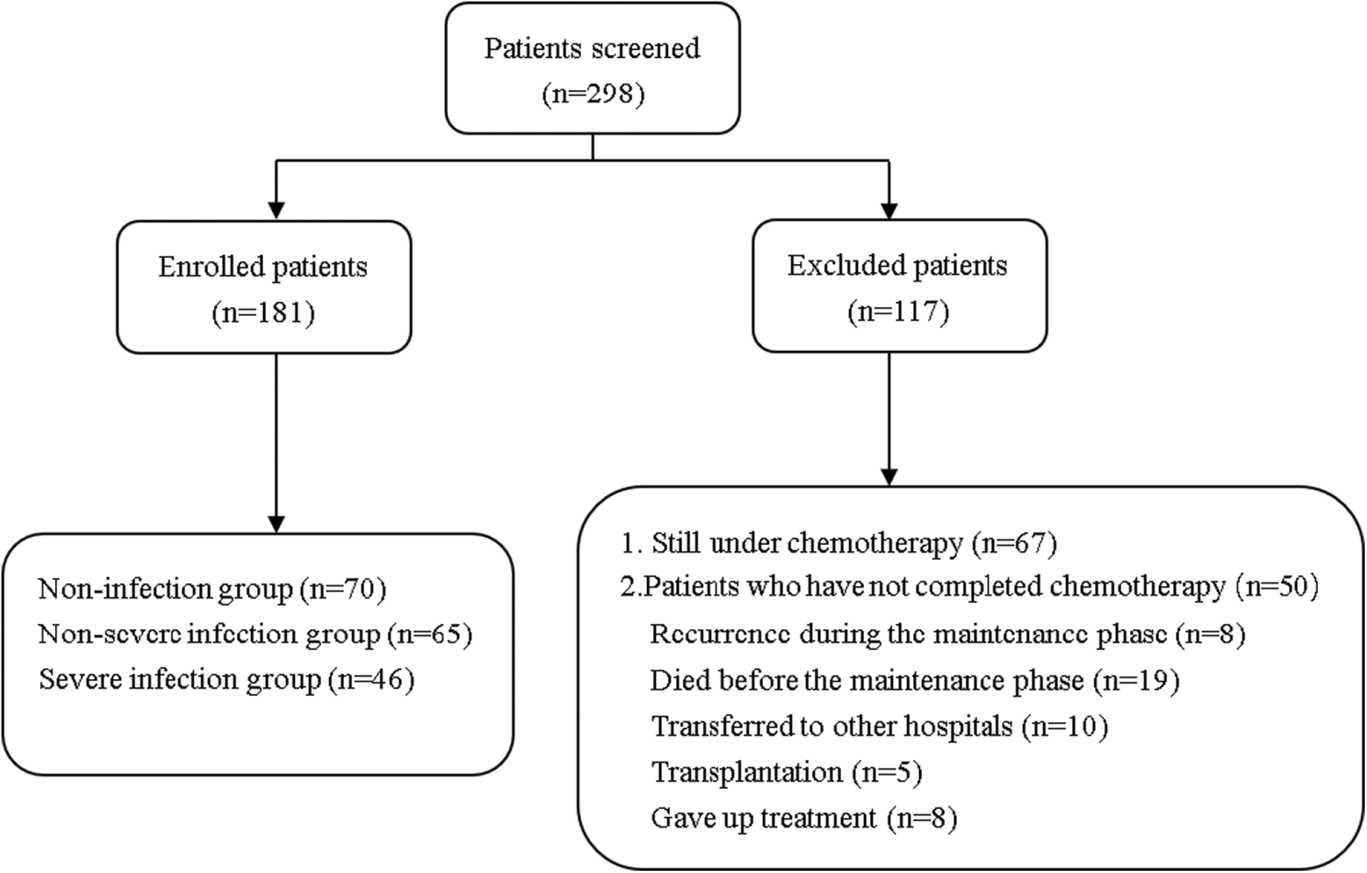
Patient enrolment flow chart.

This retrospective cohort study included patients from the Department of Pediatrics, Wuhan Union Hospital from January 1, 2015 to December 31, 2019. All patients were diagnosed with ALL and received chemotherapy according to the Chinese Children's Cancer Group ALL 2015 (CCCG‐ALL‐2015) protocol.^16^ Specific chemotherapy regimens are provided in Table S1. During the study period, a total of 298 patients enrolled in the CCCG‐ALL‐2015 protocol. As of December 1, 2022, 181 patients who completed all chemotherapy in our hospital or died of severe infection during the maintenance phase were enrolled in this study; inclusion criteria are shown in Figure 1. In addition, patients with Down syndrome were not included in this study. Ethical approval for this study was granted by the Wuhan Union Hospital Human Research Ethics Committee (approval number 2016108EP). All patients provided informed consent before participation.

### 2.2 Data collection and definition

In this study, one admission for a severe infection was defined as a severe infection event. During a single hospital stay, some patients experienced simultaneous infections in two different sites, which we considered as a severe infection event. We collected the data including gender, age, immunotyping, risk, hematological examination during maintenance treatment, and outcome of all the patients. We also collected the clinical manifestations, etiology, imaging examination, complications, anti‐infection/anti‐inflammation or supportive treatment, and hospitalization of patients with severe infection during the maintenance treatment from electronic medical records. According to the infection status during the maintenance phase, children were divided into a non‐infection group, a non‐severe infection group, and a severe infection group.

Infection groups were classified based on the CCCG‐ALL‐2015 protocol SAE criteria. The detailed criteria for severe infection in this study are as follows:

1. Pulmonary infection (any of the following exists): Pneumonia is associated with respiratory failure, ileus or gastrointestinal bleeding, shock, renal insufficiency, convulsions, or altered consciousness.
2. Digestive tract infection:
  - Patients with intestinal perforation, peritonitis, shock, or need to be transferred to the pediatric intensive care unit (PICU).
3. Other infections need to be transferred to PICU for treatment.
4. Sepsis, septic shock.
5. Other cases of severe infection, such as intracranial infection with disturbance of consciousness or convulsion.

Patients who were infected but did not meet the criteria for severe infection were classified into the non‐severe infection group.

### 2.3 Oral medication and adjustment during maintenance phase

In the CCCG‐ALL‐2015 protocol's maintenance phase, the dose of mercaptopurine is 50 mg/m^2^/day, and methotrexate is administered at a dose of 25 mg/m^2^/day. The dosage is adjusted based on the blood counts to maintain white blood cell (WBC) counts between 2 × 10^9^/L and 3.0 × 10^9^/L, absolute neutrophil count (ANC) >0.5 × 10^9^/L, and platelet counts ≥50 × 10^9^/L. Patients who do not meet these criteria cease drug intake for a week are reevaluated until their blood counts improve to continue maintenance treatment, with a 20% dosage reduction. The above criteria are doubled for patients using dexamethasone or within 48 h after its discontinuation. If the WBC count exceeds 3.5 × 10^9^/L in 3 out of 8 weeks, the dosage is increased by 20%, with a maximum additional dose of 150%.

### 2.4 Statistical analysis

Continuous variables were expressed as mean and standard deviation. Categorical variables were presented as frequency rates and percentages and were analyzed by using the chi‐squared test or Fisher's exact test as appropriate. Binary logistic regression analysis was used to identify risk factors associated with a severe infection in the maintenance phase. Statistical analysis was performed using the Statistical Package for Social Sciences version 24.0 software (SPSS Inc.). *p* < 0.05 was considered statistically significant.

## 3 RESULTS

### 3.1 Demographic data of patients

A total of 181 patients were enrolled in this study, consisting of 105 males (58%) and 76 females (42%), with an average age of 5.57 ± 2.48 years. Among the patients, 98 cases (54.1%) were low risk (LR), and 83 cases (45.9%) were intermediate or high risk (IR/HR). There were 170 (93.9%) cases of B‐ALL and 11 (6.1%) cases of T‐ALL. There were 46 patients in the severe infection group, 70 patients in the non‐infection group, and 65 patients in the non‐severe infection group. There were no significant differences among the three groups, as shown in Table 1.

**TABLE 1:** Characteristics of the 181 ALL patients.

	Non‐infection, *n* (%)	Infection, *n* (%)		Total, *n* (%)	Chi‐square	*p*‐value
		Non‐severe	Severe			
Sexy					0.985	0.611
Male	41 (58.6)	40 (61.5)	24 (52.2)	105 (58)		
Female	29 (41.4)	25 (38.5)	22 (47.8)	76 (42)		
Age					3.899	0.142
<1 year	0	0	1 (2.2)	1 (0.6)		
1–10 years	61 (87.1)	62 (95.4)	39 (84.8)	162 (89.5)		
>10 years	9 (12.9)	3 (4.6)	6 (13)	18 (9.9)		
Immunophenotyping					0.707	0.702
B‐lineage	67 (95.7)	60 (92.3)	43 (93.5)	170 (93.9)		
T‐lineage	3 (4.3)	5 (7.7)	3 (6.5)	11 (6.1)		
Risk stratification					4.555	0.103
Low risk	39 (55.7)	40 (61.5)	19 (41.3)	98 (54.1)		
Intermediate/high risk	31 (44.3)	25 (38.5)	27 (58.7)	83 (45.9)		

### 3.2 Distribution of severe infection in children with ALL during the maintenance phase

**FIGURE 2:**
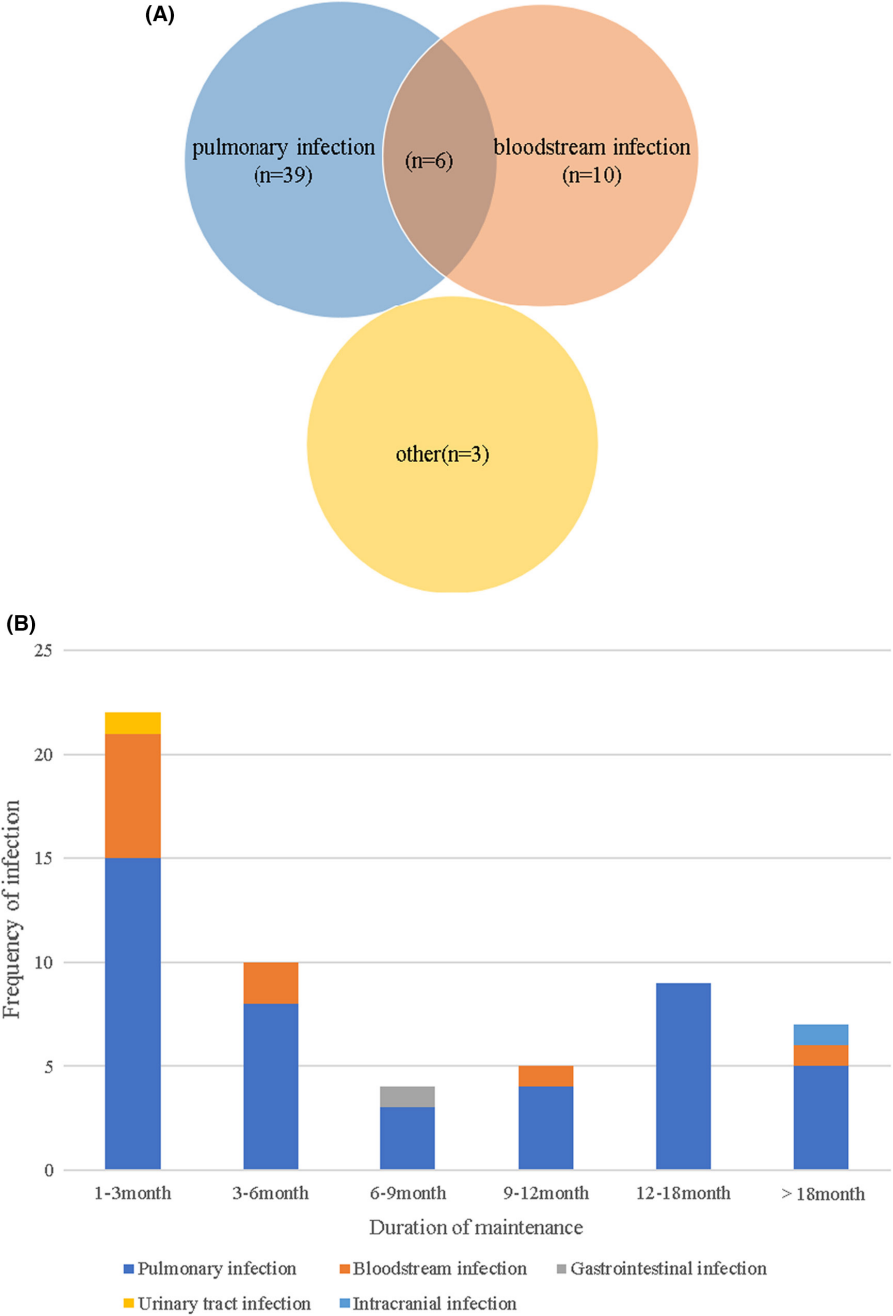
The site and distribution of severe infection in children with acute lymphoblastic leukemia (ALL) during the maintenance phase: (A) Sites of severe infection in children with ALL during the maintenance phase. Of the 46 patients, 39 had severe pulmonary infections, 10 had bloodstream infections, and three had severe infections in other sites, such as the digestive tract, urinary tract, or intracranium. Six patients had both severe lung infection and bloodstream infection. Among the 39 patients with severe pulmonary infection, 34 had severe pulmonary infection once and five had severe pulmonary infection twice. (B) Distribution of severe infection in ALL children during the maintenance phase.

Among the children with ALL in the severe infection group, 51 severe infection events occurred in 46 patients. There were various types of severe infection in ALL children during the maintenance phase, among which pulmonary infection was the most common one. Of the 46 patients, 39 had severe pulmonary infections, 10 had bloodstream infections, and three had severe infections in other sites, such as the digestive tract, urinary tract, or intracranium. Six of them had both severe pulmonary infection and bloodstream infection. Among the 39 patients with severe pulmonary infection, 34 had severe pulmonary infection once and five had severe pulmonary infection twice (Figure 2A).

Analyzing the timing of severe infections, we found that 27 events (52.9%) occurred within the first 6 months of the maintenance phase, most of which occurred in the first 3 months (17 events, 33.3%). Four events (7.8%) were reported during the first 6–9 months of the maintenance phase, while five events (9.8%) occurred during 9–12 months. During the second year of maintenance treatment, there were nine events (17.6%) and six events (11.8%) suffering from severe infection in the first and second half of the year, respectively. (Figure 2B).

### 3.3 Clinical features of ALL children with severe infection

**FIGURE 3:**
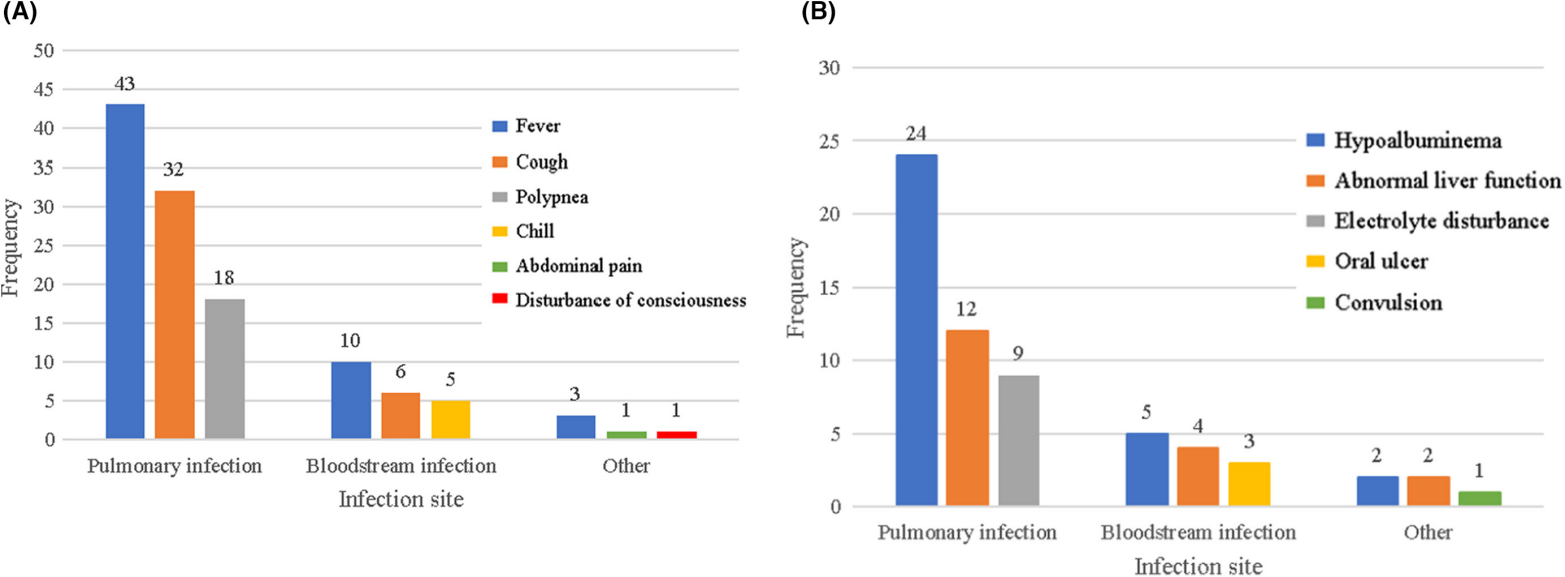
Common clinical features of acute lymphoblastic leukemia children with severe infection during the maintenance phase: (A) The common symptoms of different infection. (B) The common complications of different infection.

Fever and cough were the main symptoms of severe infection in ALL children during the maintenance phase. Among the 51 events of severe infection, fever was the most common symptom (48 events, 94.1%), followed by respiratory symptoms such as cough (35 events, 68.6%), shortness of breath (19 events, 33.9%), and expectoration (14 events, 27.5%). In addition, a few patients had weakness, abdominal pain, abdominal distension, diarrhea, fatigue, chest tightness, and other manifestations. The common symptoms associated with different types of infection are shown in Figure 3A.

In the 44 events with severe pneumonia that underwent computer tomography (CT) examination, the majority of patients exhibited ground glass opacity (25/44, 56.8%) as the predominant CT finding. Additionally, ground glass opacity and consolidation were observed in 12 events (27.3%), while streak shadow was seen in 11 events (25%). Nodules were detected in 10 events (22.7%). Pleural effusion, pleural thickening, cavity, and other manifestations were reported infrequently.

G (1,3‐β‐glucan detection) + GM test (galactomannan antigen detection) was performed in 44 of 51 severe infection events, 22 events were positive (21 events with G test positive and one event with GM test positive). Combined with the patient's medical history, serum G + GM results, imaging findings, and effective antifungal therapy, the 22 events were diagnosed as a possible fungal infection.

When severe infections occurred in children with ALL during the maintenance phase, different complications were accompanied, including serious conditions such as convulsions (3/51, 5.9%), respiratory failure (4/51, 7.8%), alveolar hemorrhage (1/51, 2%), intracranial hypertension (1/51, 2%), hydropericardium (4/51, 7.8%), hyperglycemia (1/51, 2%), and coagulopathy (2/51, 3.9%). Abnormal liver function (16/51, 31.4%), hypoalbuminemia (27/51, 52.9%), electrolyte disturbance (11/51, 21.6%), and hypothyroidism (9/51, 17.6%) were common. At the same time, skin and mucous membrane diseases were also observed, such as oral ulcer (5/51, 9.8%), thrush (3/51, 5.9%), perianal burst ulcer (3/51, 5.9%), and rash (2/56, 3.6%). The common complications of patients with different infections are shown in Figure 3B.

### 3.4 Treatment of ALL children with severe infection during the maintenance phase

The hospital stay for the severe infection group was significantly longer compared to the non‐severe infection group (16.79 ± 6.466 vs. 8.08 ± 2.451, *p* < 0.05). Among the severe infection events, 39 events (76.5%) required hospitalization for more than 2 weeks, and 12 events (23.5%) required hospitalization for 7–14 days. Among 51 events of severe infection, five events (9.8%) were transferred to PICU, four events (7.8%) progressed to shock, four events (7.8%) were given ventilator treatment, and 36 events (70.6%) were given oxygen inhalation support. Most children with severe infection were treated with multiple categories of antibacterial drugs, and 41 events (80.4%) were treated with antifungal drugs. Additionally, 34 events (66.7%) were treated with anti‐inflammatory therapy, and 14 events (27.5%) received other supportive treatments such as albumin infusions, red blood cell transfusions, or gamma globulin infusions. After combined treatment, all the children's conditions improved or recovered, and there were no death cases.

### 3.5 Risk factors for severe infection during the maintenance phase

Forty‐six ALL children with severe infection during the maintenance phase were included in the univariate analysis with variables including gender, age, immunotyping, risk, WBC count at new diagnosis, minimum neutrophil count, duration of agranulocytosis, hemoglobin, platelet levels, and globulin levels. The chi‐squared test showed that agranulocytosis, agranulocytosis ≥7 days, anemia, and hypoglobulinemia were significantly associated with a severe infection in children with ALL during the maintenance phase (*p* < 0.05) (Table 2).

**TABLE 2:** Univariate analysis of risk factors for severe infection during ALL maintenance phase.

Factors	Non‐infection	Infection		Chi‐square	*p*‐value
		Non‐severe	Severe		
	(*n* = 70)	(*n* = 65)	(*n* = 46)		
Sexy				0.985	0.611
Male	41 (58.6%)	40 (61.5%)	24 (52.2%)		
Female	29 (41.4%)	25 (38.5%)	22 (47.8%)		
Age				3.899	0.142
1–10 years	61 (87.1%)	62 (95.4%)	39 (84.8%)		
<1 year/>10 years	9 (12.9%)	3 (4.6%)	7 (15.2%)		
Immunophenotyping				0.707	0.702
B‐lineage	67 (95.7%)	60 (92.3%)	43 (93.5%)		
T‐lineage	3 (4.3%)	5 (7.7)	3 (6.5%)		
Risk stratification				4.555	0.103
Low risk	39 (55.7%)	40 (61.5%)	19 (41.3%)		
Intermediate/high risk	31 (44.3%)	25 (38.5%)	29 (58.7%)		
WBC count at initial diagnosis				4.535	0.104
<50 g/L	61 (87.1%)	54 (83.1%)	33 (71.7%)		
≥50 g/L	9 (12.9%)	11 (16.9%)	13 (28.3%)		
Minimum ANC count				35.903	**<0.0001**
<0.5 g/L	6 (8.6%)	23 (35.4%)	28 (60.9%)		
≥0.5 g/L	64 (91.4%)	42 (64.6%)	18 (39.1%)		
Agranulocytosis				27.301	**<0.0001**
<7 days	70 (100%)	61 (93.8%)	33 (71.7%)		
≥7 days	0	4 (6.2%)	13 (28.3%)		
Minimum Hb count				15.822	**<0.0001**
<90 g/L	23 (32.9%)	18 (27.7%)	29 (63%)		
≥90 g/L	47 (67.1%)	47 (72.3%)	17 (37%)		
Minimum PLT count				2.162	0.339
≤20 g/L	2 (2.9%)	1 (1.5)	3 (6.5%)		
>20 g/L	68 (97.1%)	64 (98.5%)	43 (93.5%)		
Lowest globulin level				74.852	**<0.0001**
<20 g/L	10 (14.3%)	44 (67.7%)	42 (91.3%)		
≥20 g/L	60 (85.7%)	21 (32.3%)	4 (8.7%)		

*Note*: Bold values indicates of the *p* value of < 0.05.

Furthermore, by multivariable logistic regression analysis, agranulocytosis, agranulocytosis ≥7 days, anemia, and hypoglobulinemia were identified as independent risk factors of ALL children with severe infection during the maintenance phase (*p* < 0.05) (Table 3).

**TABLE 3:** Multivariate analysis of risk factors for severe infection during acute lymphoblastic leukemia maintenance phase.

Risk factor	*β*	S.E	Wald	*p*‐value	95% CI
Agranulocytosis	2.308	0.675	11.702	**0.001**	0.986–3.631
Agranulocytosis ≥7days	−4.446	0.765	33.730	**<0.0001**	−5.946‐2.945
Anemia	−1.381	0.604	5.226	**0.022**	−2.565‐0.197
Hypoglobulinemia	2.963	0.492	36.274	**<0.0001**	1.999–3.927

*Note*: Bold values indicates of the *p* value of < 0.05.

### 3.6 The effect of severe infection on the prognosis of ALL children

The children in this study were followed up for 3–7.5 years, with an average follow‐up duration of 5.01 ± 1.16 years until July 1, 2022. In the severe infection group, 44 patients survived (95.6%), two died (4.3%) and four relapsed (8.7%). In the non‐severe infection group, 63 patients survived (96.9%), two died (3.1%) and six relapsed (9.2%). In the non‐infection group, 68 patients survived (97.1%), six relapsed (9.2%), and two died (2.9%) but not because of severe infection. None of the patients died from severe infections.

**FIGURE 4:**
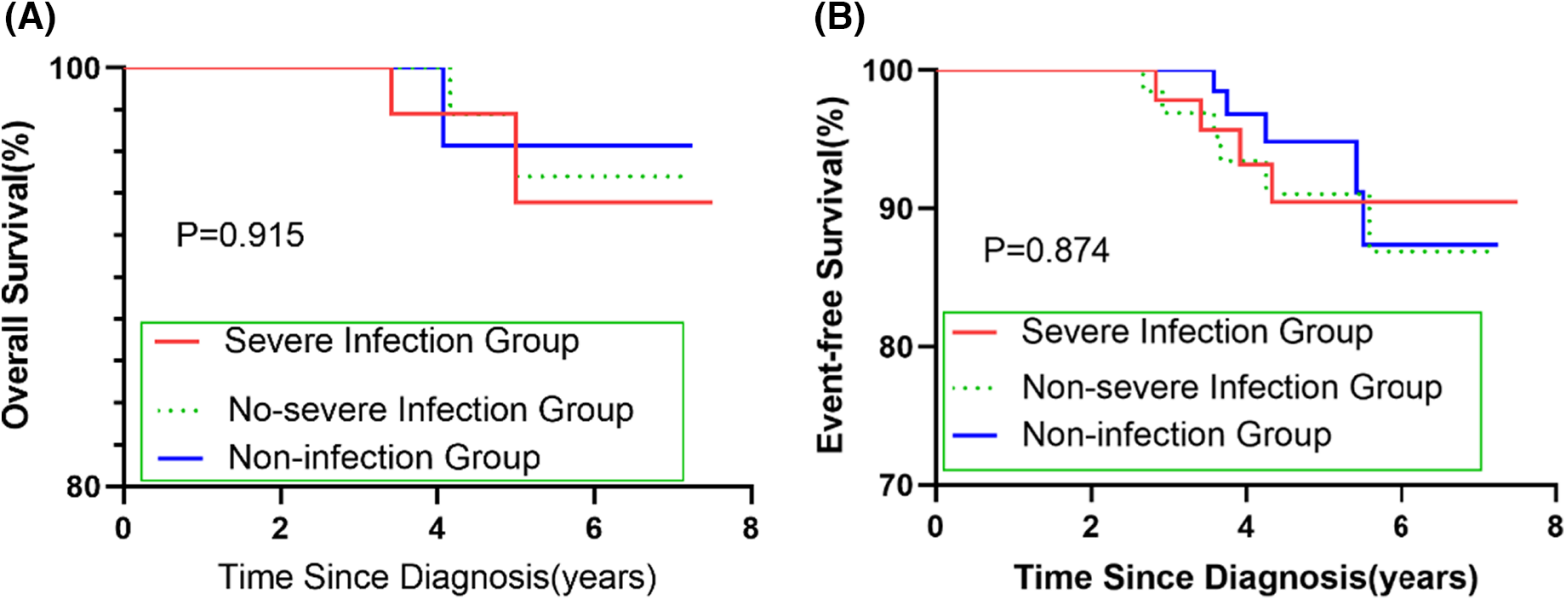
The effect of severe infection on the prognosis of acute lymphoblastic leukemia children: The 5‐year overall survival (A) and event‐free survival (B) of severe infection group, non‐severe infection group, and non‐infection group.

The 5‐year OS of the severe infection group, non‐severe infection group, and non‐infection group was 93.6 ± 0.046%, 94.8 ± 0.036%, and 96.3 ± 0.026%, respectively. The 5‐year event‐free survival (EFS) was 90.4 ± 0.046%, 91.0 ± 0.039%, and 94.8 ± 0.029%, respectively. The survival curves showed that there was no significant difference in 5‐year OS and EFS among the three groups (all *p* > 0.05, see Figure 4).

## 4 DISCUSSION

In this study, the incidence of severe infection during the ALL maintenance phase was found to be 27.6%. The occurrence time of severe infection was mainly in the first half of the year in the maintenance phase, especially in the first 3 months. It was considered that the immune injury caused by intensive chemotherapy had not recovered when the maintenance phase had just entered.^17^ The respiratory tract and bloodstream were the main sites of infection, which was consistent with the results of other studies.^8^, ^18^, ^19^ There are two reasons why respiratory tract infections are more common in children with ALL. First, compared to adults, children have narrower nasal passages, shorter and narrower trachea and bronchus, fragile mucosa, less mucinous gland secretion, poor ciliary swing ability, and poor non‐specific and specific immune function. Second, chemotherapy can cause systemic mucosal damage, especially of the oral cavity and respiratory tract, colonization by various pathogens, and then infection.^20^ Pathogens enter the blood easily through damaged mucous membrane, so bloodstream infection is also commonly seen.

Pneumonia often shows typical features in children without ALL, but children with ALL present atypical clinical manifestations because of immune dysfunction.^21^, ^22^ In this study, the most common clinical manifestations in children complicated with pulmonary infection were fever, cough, and shortness of breath, with no obvious changes in physical signs. Most of the patients rapidly developed dyspnea or even respiratory failure and other serious complications. Therefore, early detection and treatment are crucial in order to control disease progression and improve prognosis. However, sometimes early diagnosis of pneumonia is difficult, which requires comprehensive diagnosis based on imaging and etiology.^23^ Chest radiography is commonly tested in normal children with suspected pneumonia, but researchers believe that chest radiography plays a limited role in the early evaluation of pulmonary infection in children with ALL. Instead, pulmonary CT examination is strongly recommended in these cases. In this study, the imaging of children with pneumonia was mainly characterized by the ground glass shadow, consolidation shadow, streak shadow, and nodule shadow, which suggested bacterial and/or fungal infection.^24^, ^25^

Bloodstream infection was more difficult to diagnose, because the positive rate of blood culture was low—only 19.6% in the severe infection group. The time of blood collection, the number of blood samples, and technology all influenced blood culture results. Not all the blood samples were taken when the patients were suffering from fever or chills. And some samples were collected after using antibiotics. As regards the young age, it was difficult to obtain sufficient blood culture samples or repeated examinations. What is more, conventional blood cultures have a limited positive detection rate. In order to increase the positive detection rate of etiology, we should actively send double samples of blood culture or choose high‐throughput sequencing.

In this study, the pathogenic bacteria detected by blood culture were mainly Gram‐positive bacteria. In the past, many studies have shown that Gram‐negative bacteria were the primary cause of infection in children with ALL during chemotherapy.^26^ However, in recent years, with the popularization of peripherally inserted central catheter (PICC) and infusion ports, the proportion of Gram‐positive bacteria infection in ALL patients is increasing. Additionally, fungal infection is also commonly observed in ALL children due to bone marrow suppression and suppressed immunity.^27^ Some studies report that fungal infections account for about 20% of infection‐related deaths.^26^ Prophylactic application of antibacterial and antifungal drugs has been shown to reduce the incidence of infection. It was reported that levofloxacin prophylaxis significantly reduced febrile neutrophil, bacteremia, and clinically documented infection. Furthermore, the use of broad‐spectrum antibiotics did not result in a higher incidence of adverse events, such as fungal infections or drug‐resistant bacteria.^28^ Prophylaxis with ciprofloxacin, voriconazole, or micafungin could reduce the incidence of bloodstream infections and invasive fungal infections in children with acute leukemia receiving intensive chemotherapy, as well as the onset of febrile neutropenia and the length of ICU stay.^29^ Sulfamethoxazole and trimethoprim are also recommended for use prophylactically in ALL patients from induction to the end of maintenance chemotherapy.^30^, ^31^

ALL children complicated with severe infection have a serious disease, rapid progression, and are prone to suffer from septic shock and multiple organ dysfunction leading to death. In the past, there have been many studies on risk factors related to infection during the induction of chemotherapy for ALL patients and it is believed that Down syndrome, agranulocytosis, long‐term agranulocytosis, skin mucosal damage, and hypoalbuminemia are risk factors for infection during induction of chemotherapy.^32^, ^33^ At present, there are few studies on the risk factors related to infection in the maintenance phase of ALL. Ning et al. have shown that Down syndrome, high Patient‐Generated Subjective Global Assessment (PG‐SGA) score, intense chemotherapy, agranulocytosis, low level of hemoglobin or albumin, and long‐term use of antibiotics are risk factors of infection in the maintenance phase of ALL children.^34^ Our study found that agranulocytosis, agranulocytosis ≥7 days, anemia, and hypoglobulinemia were independent risk factors for severe infection during the maintenance phase.

Since 1977, Bodey and his colleagues have proposed that a low ANC is associated with infection. Agranulocytosis and its duration have been widely considered as risk factors for cancer infection.^23^, ^35^ The long‐term and high‐dose administration of chemotherapy drugs and their metabolites can directly damage the bone marrow microenvironment and bone marrow progenitor cells, leading to neutropenia and even agranulocytosis.^36^ Although the intensity of chemotherapy during the maintenance phase is relatively low, a small number of patients may still experience severe myelosuppression due to genetic polymorphism associated with 6‐mercaptopurine. Therefore, regular follow‐up and adjustment of medication under the guidance of doctors are necessary. Granulocyte colony‐stimulating factor stimulates the production of neutrophils and increases the migration capacity and production of superoxide, which helps monocyte‐directed cellular immunity and can be used to treat patients with agranulocytosis and aggressive fungal infections.^37^ In vitro results showed that granulocytes retained function and rapidly migrated to the site of infection despite a short half‐life.^38^ Successful outcomes have been reported for other invasive mycoses or severe infection when patients with profound neutropenia were supported with granulocyte transfusions.^39^, ^40^, ^41^, ^42^, ^43^ Anemia can damage a child's immune system and exacerbate the risk of infections. There is evidence suggesting that red blood cells are directly involved in the maintenance of innate and adaptive immune systems and are modulators of T‐cell proliferation.^44^ Consequently, children with anemia are more susceptible to develop serious infections.

Although the immune function of children in the maintenance phase of ALL partially recovers, it remains relatively low, and the ability to resist infection was poor. Immunoglobulins and T and B lymphocytes are the main components of the body's immune defense mechanism, and previous studies have suggested that the abnormalities in the function and quantity of these cells are associated with infections in children with ALL. ALL patients are prone to infection due to the low level of immunoglobulin due to the abnormal function of B cells. This study also confirmed that the level of globulin is a risk factor for infection. El‐Chennawi et al, observed that low levels of CD19, IgM, and CD4^+^/CD8^+^ T‐cell ratios were associated with infection in children with ALL during the maintenance phase.^14^ Ateyah et al. found that high levels of Treg cells were associated with Epstein–Barr virus infection in ALL patients.^45^ However, there are still discrepancies in the findings regarding gender, age, and risk stratification as risk factors for infection, which need further research to explore. In clinical work, children with high risk factors of severe infection can be intervened and treated as early as possible.

This study still has some limitations. It is a single‐center retrospective study with a relatively limited sample size, and the results of different regions and countries may be different due to differences in medical conditions, economy, and other aspects. In conclusion, blood routine, lymphoid subsets, and immunoglobulin levels should be monitored regularly for ALL patients during the maintenance phase, especially in the first 6 months. For ALL children with agranulocytosis, agranulocytosis ≥7 days, anemia, and low globulin levels, preventive anti‐infection therapy or supportive therapy can be given as appropriate to reduce the occurrence of severe infection. However, the impact of prophylactic therapy on maintenance therapy still needs further investigation.

## AUTHOR CONTRIBUTIONS

**Tiantian Yin:** Conceptualization (equal); data curation (lead); formal analysis (lead); investigation (lead); methodology (lead); project administration (equal); resources (lead); software (lead); supervision (supporting); validation (lead); visualization (lead); writing – original draft (lead). **Juan Han:** Data curation (equal); project administration (equal); writing – review and editing (equal). **Jinjin Hao:** Data curation (equal); supervision (equal); writing – review and editing (supporting). **Hui Yu:** Data curation (equal); validation (equal); writing – review and editing (supporting). **Yining Qiu:** Data curation (equal); supervision (equal); writing – review and editing (supporting). **Jiawei Xu:** Data curation (equal); supervision (equal); writing – review and editing (supporting). **Yun Peng:** Data curation (equal); supervision (equal); writing – review and editing (supporting). **Xiaoyan Wu:** Data curation (equal); supervision (equal); writing – review and editing (supporting). **Runming Jin:** Conceptualization (equal); formal analysis (equal); funding acquisition (equal); project administration (equal); supervision (equal); visualization (equal); writing – review and editing (lead). **Fen Zhou:** Conceptualization (lead); formal analysis (equal); funding acquisition (lead); methodology (lead); project administration (lead); resources (equal); supervision (lead); visualization (equal); writing – review and editing (lead).

## FUNDING INFORMATION

This study is funded by the National Natural Science Foundation of China (No. 82070172).

## CONFLICT OF INTEREST STATEMENT

The authors have no relevant financial or nonfinancial interests to disclose.

## ETHICS STATEMENT

This study was approved by the Wuhan Union Hospital Human Research Ethics Committee (approval number 2016108EP) and informed consent was obtained from the parents, guardians, or patients, as appropriate, before participation.

## Supporting information


Table S1:

## Data Availability

The data generated for this study are available from the corresponding author upon reasonable request.

## References

[1] Kato M , Manabe A . Treatment and biology of pediatric acute lymphoblastic leukemia. Pediatr Int. 2018;60(1):4‐12. 29143423 10.1111/ped.13457

[2] Steliarova‐Foucher E , Colombet M , Ries LAG , et al. International incidence of childhood cancer, 2001‐10: a population‐based registry study. Lancet Oncol. 2017;18(6):719‐731. 28410997 10.1016/S1470-2045(17)30186-9 PMC5461370

[3] Inaba H , Greaves M , Mullighan CG . Acute lymphoblastic leukaemia. Lancet. 2013;381(9881):1943‐1955. 23523389 10.1016/S0140-6736(12)62187-4 PMC3816716

[4] Pui CH , Yang JJ , Bhakta N , Rodriguez‐Galindo C . Global efforts toward the cure of childhood acute lymphoblastic leukaemia. Lancet Child Adolesc Health. 2018;2(6):440‐454. 30169285 10.1016/S2352-4642(18)30066-X PMC6467529

[5] Gaudichon J , Jakobczyk H , Debaize L , et al. Mechanisms of extramedullary relapse in acute lymphoblastic leukemia: reconciling biological concepts and clinical issues. Blood Rev. 2019;36:40‐56. 31010660 10.1016/j.blre.2019.04.003

[6] Yao JF , Li N , Jiang J . Clinical characteristics of bloodstream infections in pediatric acute leukemia: a single‐center experience with 231 patients. Chin Med J (Engl). 2017;130(17):2076‐2081. 28836551 10.4103/0366-6999.213411 PMC5586176

[7] Ozturk AP , Koc B , Zulfikar B . Acute complications and survival analysis of childhood acute lymphoblastic leukemia: a 15‐year experience. Clin Lymphoma Myeloma Leuk. 2021;21(1):e39‐e47. 33046422 10.1016/j.clml.2020.08.025

[8] Inaba H , Pei D , Wolf J , et al. Infection‐related complications during treatment for childhood acute lymphoblastic leukemia. Ann Oncol. 2017;28(2):386‐392. 28426102 10.1093/annonc/mdw557 PMC5834143

[9] Oh BLZ , Fan L , Lee SHR , et al. Life‐threatening infections during treatment for acute lymphoblastic leukemia on the Malaysia‐Singapore 2003 and 2010 clinical trials: a risk prediction model. Asia Pac J Clin Oncol. 2022;18(5):e456‐e468. 35134276 10.1111/ajco.13756

[10] Teachey DT , Hunger SP , Loh ML . Optimizing therapy in the modern age: differences in length of maintenance therapy in acute lymphoblastic leukemia. Blood. 2021;137(2):168‐177. 32877503 10.1182/blood.2020007702 PMC7820874

[11] Schmiegelow K , Nielsen SN , Frandsen TL , Nersting J . Mercaptopurine/methotrexate maintenance therapy of childhood acute lymphoblastic leukemia: clinical facts and fiction. J Pediatr Hematol Oncol. 2014;36(7):503‐517. 24936744 10.1097/MPH.0000000000000206 PMC4222610

[12] Terwilliger T , Abdul‐Hay M . Acute lymphoblastic leukemia: a comprehensive review and 2017 update. Blood Cancer J. 2017;7(6):e577. 28665419 10.1038/bcj.2017.53 PMC5520400

[13] Toksvang LN , Lee SHR , Yang JJ , Schmiegelow K . Maintenance therapy for acute lymphoblastic leukemia: basic science and clinical translations. Leukemia. 2022;36(7):1749‐1758. 35654820 10.1038/s41375-022-01591-4 PMC9252897

[14] El‐Chennawi FA , Al‐Tonbary YA , Mossad YM , Ahmed MA . Immune reconstitution during maintenance therapy in children with acute lymphoblastic leukemia, relation to co‐existing infection. Hematology. 2008;13(4):203‐209. 18796245 10.1179/102453308X316086

[15] Luczynski W , Stasiak‐Barmuta A , Krawczuk‐Rybak M . Immunologic monitoring of maintenance therapy for acute lymphoblastic leukaemia in children‐preliminary report. Pediatr Blood Cancer. 2004;42(5):416‐420. 15049012 10.1002/pbc.20018

[16] Cai J , Yu J , Zhu X , et al. Treatment abandonment in childhood acute lymphoblastic leukaemia in China: a retrospective cohort study of the Chinese Children's cancer group. Arch Dis Child. 2019;104(6):522‐529. 30705079 10.1136/archdischild-2018-316181

[17] Lovat PE , Robinson JH , Windebank KP , Kernahan J , Watson JG . Serial study of T lymphocytes in childhood leukemia during remission. Pediatr Hematol Oncol. 1993;10(2):129‐139. 8318367 10.3109/08880019309016546

[18] Bakhshi S , Padmanjali KS , Arya LS . Infections in childhood acute lymphoblastic leukemia: an analysis of 222 febrile neutropenic episodes. Pediatr Hematol Oncol. 2008;25(5):385‐392. 18569840 10.1080/08880010802106564

[19] Mairuhu AM , Andarsini MR , Setyoningrum RA , et al. Hospital acquired pneumonia risk factors in children with acute lymphoblastic leukemia on chemotherapy. Heliyon. 2021;7(6):e07209. 34169164 10.1016/j.heliyon.2021.e07209 PMC8207214

[20] Christensen MS , Heyman M , Mottonen M , et al. Treatment‐related death in childhood acute lymphoblastic leukaemia in the Nordic countries: 1992‐2001. Br J Haematol. 2005;131(1):50‐58. 16173962 10.1111/j.1365-2141.2005.05736.x

[21] Freifeld AG , Bow EJ , Sepkowitz KA , et al. Clinical practice guideline for the use of antimicrobial agents in neutropenic patients with cancer: 2010 update by the Infectious Diseases Society of America. Clin Infect Dis. 2011;52(4):427‐431. 21205990 10.1093/cid/ciq147

[22] Kotloff RM , Ahya VN , Crawford SW . Pulmonary complications of solid organ and hematopoietic stem cell transplantation. Am J Respir Crit Care Med. 2004;170(1):22‐48. 15070821 10.1164/rccm.200309-1322SO

[23] Wong JL , Evans SE . Bacterial pneumonia in patients with cancer: novel risk factors and management. Clin Chest Med. 2017;38(2):263‐277. 28477638 10.1016/j.ccm.2016.12.005 PMC5424613

[24] Voulgaridou A , Athanasiadou KI , Athanasiadou E , Roilides E , Papakonstantinou E . Pulmonary infectious complications in children with hematologic malignancies and chemotherapy‐induced neutropenia. Diseases. 2020;8(3):32. 32824956 10.3390/diseases8030032 PMC7564221

[25] Nambu A , Ozawa K , Kobayashi N , Tago M . Imaging of community‐acquired pneumonia: roles of imaging examinations, imaging diagnosis of specific pathogens and discrimination from noninfectious diseases. World J Radiol. 2014;6(10):779‐793. 25349662 10.4329/wjr.v6.i10.779 PMC4209424

[26] O'Connor D , Bate J , Wade R , et al. Infection‐related mortality in children with acute lymphoblastic leukemia: an analysis of infectious deaths on UKALL2003. Blood. 2014;124(7):1056‐1061. 24904116 10.1182/blood-2014-03-560847

[27] Zawitkowska J , Drabko K , Szmydki‐Baran A , et al. Infectious profile in children with ALL during chemotherapy: a report of study group for infections. J Infect Chemother. 2019;25(10):774‐779. 31101529 10.1016/j.jiac.2019.04.005

[28] Wolf J , Tang L , Flynn PM , et al. Levofloxacin prophylaxis during induction therapy for pediatric acute lymphoblastic leukemia. Clin Infect Dis. 2017;65(11):1790‐1798. 29020310 10.1093/cid/cix644 PMC5850441

[29] Yeh TC , Liu HC , Hou JY , et al. Severe infections in children with acute leukemia undergoing intensive chemotherapy can successfully be prevented by ciprofloxacin, voriconazole, or micafungin prophylaxis. Cancer. 2014;120(8):1255‐1262. 24415457 10.1002/cncr.28524

[30] Mantadakis E . *Pneumocystis jirovecii* pneumonia in children with hematological malignancies: diagnosis and approaches to management. J Fungi (Basel). 2020;6(4):331. 33276699 10.3390/jof6040331 PMC7761543

[31] Maschmeyer G , Helweg‐Larsen J , Pagano L , et al. ECIL guidelines for treatment of pneumocystis jirovecii pneumonia in non‐HIV‐infected haematology patients. J Antimicrob Chemother. 2016;71(9):2405‐2413. 27550993 10.1093/jac/dkw158

[32] Afzal S , Ethier MC , Dupuis LL , et al. Risk factors for infection‐related outcomes during induction therapy for childhood acute lymphoblastic leukemia. Pediatr Infect Dis J. 2009;28(12):1064‐1068. 19773675 10.1097/INF.0b013e3181aa6eae

[33] Zhao JO , Yuan YF , Zhang RR , et al. Risk factors of nosocomial infection in children with acute lymphoblastic leukemia during induction and remission chemotherapy. West China Medical Science. 2019;34(4):370‐374.

[34] Ning FY , Lan JP , Chen YR , et al. Relationship between nutritional status and the incidence of nosocomial infection in patients with high‐risk acute lymphoblastic leukemia during maintenance treatment. Chinese General Practice. 2017;15(9):1582‐1585.

[35] Carvalho AS , Lagana D , Catford J , Shaw D , Bak N . Bloodstream infections in neutropenic patients with haematological malignancies. Infect Dis Health. 2020;25(1):22‐29. 31586572 10.1016/j.idh.2019.08.006

[36] Ammann RA , Laws HJ , Schrey D , et al. Bloodstream infection in paediatric cancer centres—leukaemia and relapsed malignancies are independent risk factors. Eur J Pediatr. 2015;174(5):675‐686. 25804192 10.1007/s00431-015-2525-5

[37] Erker C , Huppler AR , Walsh TJ , et al. Successful treatment of invasive Conidiobolus infection during therapy for acute lymphoblastic leukemia. J Pediatr Hematol Oncol. 2018;40(7):e446‐e449. 28991126 10.1097/MPH.0000000000000985 PMC5904005

[38] Bashir S , Stanworth S , Massey E , Goddard F , Cardigan R . Neutrophil function is preserved in a pooled granulocyte component prepared from whole blood donations. Br J Haematol. 2008;140(6):701‐711. 18302716 10.1111/j.1365-2141.2008.06996.x

[39] Garg A , Gupta A , Mishra A , Singh M , Yadav S , Nityanand S . Role of granulocyte transfusions in combating life‐threatening infections in patients with severe neutropenia: experience from a tertiary care Centre in North India. PloS One. 2018;13(12):e0209832. 30589898 10.1371/journal.pone.0209832 PMC6307785

[40] Netelenbos T , Massey E , de Wreede LC , et al. The burden of invasive infections in neutropenic patients: incidence, outcomes, and use of granulocyte transfusions. Transfusion. 2019;59(1):160‐168. 30383912 10.1111/trf.14994 PMC7379528

[41] West KA , Gea‐Banacloche J , Stroncek D , Kadri SS . Granulocyte transfusions in the management of invasive fungal infections. Br J Haematol. 2017;177(3):357‐374. 28295178 10.1111/bjh.14597 PMC5403628

[42] Liu CY , Li C . Clinical features and risk factors of severe pneumonia in children with acute lymphoblastic leukemia. Front Pediatr. 2022;10:813638. 35601429 10.3389/fped.2022.813638 PMC9120655

[43] Sachs UJ , Reiter A , Walter T , et al. Safety and efficacy of therapeutic early onset granulocyte transfusions in pediatric patients with neutropenia and severe infections. Transfusion. 2006;46(11):1909‐1914. 17076845 10.1111/j.1537-2995.2006.00996.x

[44] Gelaw Y , Getaneh Z , Melku M . Anemia as a risk factor for tuberculosis: a systematic review and meta‐analysis. Environ Health Prev Med. 2021;26(1):13. 33485299 10.1186/s12199-020-00931-z PMC7824931

[45] Ateyah ME , Hashem ME , Abdelsalam M . Epstein‐Barr virus and regulatory T cells in Egyptian paediatric patients with acute B lymphoblastic leukaemia. J Clin Pathol. 2017;70(2):120‐125. 27458150 10.1136/jclinpath-2016-203803

