# Supplementary material for: Clinical characteristics and risk factors of acute lymphoblastic leukemia in children with severe infection during maintenance treatment
Source: Cancer Med. 2023 Sep 28;12(19):19372–82. doi: 10.1002/cam4.6495 (PMC10587982; doi:10.1002/cam4.6495)
Supplement: Supplementary file 1 — Table S1: [file CAM4-12-19372-s001.docx]

Supplementary table 1: CCCG-ALL-2015 Protocol Treatment Plan Framework for Different Risk Stratification

| Risk stratification | Induction therapy  1-4 week | CAT  4-7 week | HD-MTX  8-15 week | Continue the therapy  16-31 week | Reinduction  32-34 week | Maintenance  35-54 week | Randomization maintenance  55-109 week | Maintenance  111-118 week |
| --- | --- | --- | --- | --- | --- | --- | --- | --- |
| LR | PVDL | 3 weeks in total | 3g/m^2^,  4 times | DVDL | DVL | 6MP+MTX and DVM | A or B group | 6MP+MTX |
| IR/HR | PVDL | 3 weeks in total | 5g/m^2^,  4 times | DVDL | HD-Arac | 6MP+MTX and DVCA | A or B group | 6MP+MTX |

Note: CCCG-ALL-2015 was selected by the Pediatric Cancer Committee of Chinese Anti-Cancer Association for acute lymphoblastic leukemia 2015 study: CAT is cyclophosphamide + cytarabine +6-MP. HD-MTX is a high-dose methotrexate. PVDL is prednisone + vincristine + daunorubicin + asparaginase. DVDL consists of dexamethasone + vincristine + daunorubicin + asparaginase. DVL is dexamethasone + vincristine + asparaginase. HD-Arac is a high-dose cytarabine. 6-MP is 6 mercaptopurine. MTX is methotrexate. DVM is dexamethasone + vincristine +6-MP. DVCA is dexamethasone + Vincristine + cyclophosphamide + cytarabine. Group A: dexamethasone and vincristine were added to the last 7 cycles of maintenance therapy; Group B: Dexamethasone and vincristine were not added for the last 7 cycles of treatment. The difference between LR and IR/HR group was as follows: the LR group received 16 times of intravaginal injection, the total dose of daunorubicin was 75mg/m^2^, and pemasdong was administered 3 times. In the IR/HR group, 20 times of sheath injection, the total dose of daunorubicin 175mg/m^2^, 9 times of pemasdong; "Once the BCR-ABL fusion gene is positive, randomization with dasatinib or imatinib is performed immediately; The HR group should receive allogeneic transplantation therapy after completion of HD-MTX, otherwise the same as the IR group.
